# The rhythm of chemotherapy and cancer patients’ time perspectives

**DOI:** 10.7717/peerj.14486

**Published:** 2022-12-14

**Authors:** Marcin Moskalewicz, Piotr Kordel, Anna Sterna

**Affiliations:** 1 Philosophy and Mental Health Unit, Department of Social Sciences and the Humanities, Poznań University of Medical Sciences, Poznań, Wielkopolskie, Poland; 2 Institute of Philosophy, Maria Curie-Sklodowska University Lublin, Lublin, Lubelskie, Poland

**Keywords:** Temporal experience, Chemotherapy, Zimbardo Time Perspective Inventory, Transcendental-Future Time Perspective Inventory

## Abstract

**Background:**

While it is well known that illnesses such as cancer modify the experience of time, the impact of the rhythm and length of treatment on patients’ time perspectives remains unknown.

**Methods:**

A short version of Zimbardo Time Perspective Inventory and Transcendental Future Perspective Questionnaire as well as a demographic questionnaire on a convenience sample of 259 patients (66.8% female, mean age 52.36) with various cancers and undergoing chemotherapy with different frequencies (1, 2, 3 weeks) and mean time in treatment 23.4 months.

**Results:**

The temporal perspectives mean scores of cancer patients are: positive past 3.69, negative past 3.13, present hedonism 3.08, future 3.77, transcendental future 3.40. Patients tend only slightly to lose faith alongside the course of oncological treatment regardless of their age (*ρ* =  − 0.210, *p* < 0.01). The frequency of chemotherapy mildly differentiates temporal perspectives of patients regarding present hedonism and transcendental future: a weekly treatment is more disturbing than the triweekly one and no treatment in terms of hedonism, while patients not in chemo score significantly higher in transcendental future than patients in biweekly and triweekly chemo.

**Conclusions:**

The variations of treatment rhythm are less significant than predicted, although still relevant. Since most sociodemographic variables are of no relevance, cancer experience likely unifies temporal perspectives among people of different backgrounds.

## Introduction

The phenomenon of time can be conceptualized in several ways. Researchers typically distinguish linear clock-time, the subjective experience of past, present, and future, and circular temporality (represented by *e.g.*, changing seasons or circadian rhythms). Since 1999, Zimbardo and Boyd have been developing a model of the so-called time perspectives (Zimbardo & Boyd, 1999; Zimbardo & Boyd, 2008; Zimbardo & Boyd, 2015). In that approach, the focus is not on the subjective perception of an objective time passage, nor on the recurrence of events, but on the experiential contents related to the temporal categories of past, present, and future. Based on a psychological test called Zimbardo Time Perspective Inventory (ZTPI), these contents are secondarily epistemically framed as negative past, positive past, hedonistic present, fatalistic present, future, and transcendental future. The experiential data from which our judgments and plans originate thus belongs to particular time frames.

While reasoning and planning one’s actions, people have the dispositions to rely on a particular time frame. For example, while planning future goals and strategies, some set their negative past events as a starting point, while others base their plans on positive past events. In consequence, depending on the dominant temporal perspective, the content of future plans and approaches towards them changes. Hence, temporal perspectives emerge as a powerful precognitive tool that affects one’s emotional outlook, decision-making, and actions. Time frames conceptualized in ZTPI also impact one’s adaptation to critical events, such as serious illness. Temporal perspectives were found to be a significant determinant of illness acceptance (Zawadzka & Byrczek, 2012; Król et al., 2015), life quality in the course of illness (Faury et al., 2019), diagnostic decisions made, such as genetic tests (Wakefield et al., 2010) and screening (Roncancio, Ward & Fernandez, 2014). Moreover, the dominant temporal perspectives differ between patients suffering from various diseases (Nozari, Janbabai & Dousti, 2013). Unfortunately, as far as cancer is concerned, research is limited. Positive past and hedonistic present perspectives were linked with the use of more adaptive stress-coping strategies in the course of cancer (Furmańska et al., 2019). A tendency to rely on positive past was observed among women with cancer undergoing combined treatment (Kroyan, Gurova & Ippolitova, 2019).

Recent studies proved the ZTPI model to have great structural validity (Peng et al., 2021), and be widely replicable, *e.g.*, in Tukey (Akirmak, 2021), United Kingdom (Worrell, McKay & Andretta, 2015), Sweden (Carelli, Wiberg & Wiberg, 2011), which makes it relevant for exploring cultural variations (Sircova et al., 2014). In addition, due to its vide application to many social and clinical populations worldwide, ZTPI has the great advantage of delivering standardized knowledge on time perspectives that is suitable for further comparative research (Stolarski, Fieulaine & Van Beek, 2015).

Qualitative studies’ results, on the other hand, although less comparable, bring more insights into lived experience. As far as cancer is concerned, it was found that incurable disease shortens one’s temporal horizon (Ellingsen et al., 2013) and that advanced cancer may provoke spiritual pain and impose the sense of meaninglessness and worthlessness of life (Tamura, Kikui & Watanabe, 2006). Because of its traumatic potential, the cancer experience interrupts the temporal continuity and coherence of the patient’s life (De Luca Picione, Luisa Martino & Freda, 2017). Cancer induces the need for reformulation of the previous attitude towards oneself.

A key finding of a recent phenomenological qualitative study of ovarian cancer patients concerned the so-called chemo-clock (Moskalewicz, Popova & Wiertlewska-Bielarz, 2021; Moskalewicz, Popova & Wiertlewska-Bielarz, 2022). It was found that the patients use the triweekly treatment scheme of their chemotherapy sessions to measure the passing of time—in other words, the mere rhythm of chemotherapy largely affects their temporal experience. However, there is no comparable quantitative research, and it is not known how the length of chemotherapy and its various rhythms impact temporal experience in the cancer population.

Following the previous finding, this study hypothesizes that there is a relationship between, on the one hand, linear time as measured by the length of treatment and circular time as defined by the changing rhythms of treatment, and, on the other hand, the content of patients’ experiences concerning their past, present, and future. Its aim is to reveal how the linear and circular temporality of cancer treatment translates into temporal experience objectified by ZTPI. In this way, this study aims to fill the gap in understanding the nature of temporal framing in the course of cancer treatment. Doing so may help to determine both possible psychological facilitators of treatment and the means of fostering the process of mental adjustment to the demands of recovery.

## Materials & Methods

This cross-sectional study used a simplified version of ZTPI validated for the Polish language and found to be a valid and reliable instrument (Przepiorka, Sobol-Kwapinska & Jankowski, 2016) as well as the Transcendental-Future Time Perspective Questionnaire (TTPI), which is a ZTPI supplementary scale used to asses one’s attitudes towards future after death (Boyd & Zimbardo, 1997). Both questionnaires were accompanied by demographic questions (age, sex, education, place of residence, children, income, religion, type of cancer) as well as questions concerning linear and circular time (time elapsed since diagnosis and the beginning of treatment, and the current frequency of chemotherapy). These latter questions concerning temporality were crucial from the perspective of the study hypotheses, and the data on linear and circular time was later treated as independent variables for determining its impact on the time perspectives of patients.

Data was collected at chemotherapy wards of the Maria Skłodowska–Curie Greater Poland Cancer Centre in Kalisz, the Oncological Wards of Voivodship Hospital in Konin, and Pleszew Medical Center (all in Poland). A convenience sampling method was used: all patients who had been receiving chemotherapy for at least a month and were present at the wards when the data were collected were asked to fill in the survey. Poznan University of Medical Sciences IRB approved the study as a non-experimental type (decision from 08.09.2020). All respondents were assured of anonymity and gave their verbal informed consent for participation.

The final sample consisted of 259 cancer patients: 66.8% female and 33.2% male, with a mean age of 52.36 years (SD 16.49), mean time passed since diagnosis of 32.23 months (min.-max. 1-840, Me 12.00, SD 67.42) and 23.4 months mean time since the beginning of chemo (min.-max. 1-204, Me 12.00, SD 32.79). The patients had the following cancers: breast 32.4%, colon 17.4%, lung 8.5%, prostate 6.9%, ovarian 4.6%, cervical 3.5%, skin 2.7%, lymphoma 9.3%, other 10.1%. The group was therefore highly diverse regarding cancer type and duration of illness. As far as the treatment rhythm is concerned, 12.4% (*n* = 32) of the sample currently had their chemotherapy sessions once a week, 26.3% (*n* = 68) once every two weeks, and 29.0% (*n* = 75) once every three weeks; while 32.4% (*n* = 84) had recently finished treatment. The ZTPI and the TTPI Cronbach’s alphas were: .725 for Past Negative, .745 for Present Hedonistic, .773 for Future, .691 for Past Positive, and .873 for Transcendental Future, which is close to the results for Polish general population (Przepiorka, Sobol-Kwapinska & Jankowski, 2016).

The study aimed to find how the length of treatment and the frequency of chemotherapy sessions affects patients’ temporal perspectives as measured by the ZTPI and TTPI. Since the Kolmogorov–Smirnov test showed a lack of normal distribution, *χ*2, Kruskal-Wallis H and Mann–Whitney *U* tests were used; correlations were calculated using Spearman’s *ρ* (alpha = 0.05). All the tests were run using IBM SPSS Statistics v.22 software (IBM, Chicago, IL, USA).

## Results

Basic demographic data: 79.9% (*n* = 207) of the patients declared to be religious (all catholic due to Polish cultural context), the rest identified themselves as nonbelievers; the majority of the studied group was in a relationship (62.2%, *n* = 161), 15.8% (*n* = 41) were single, 9.3% (*n* = 24) divorced, and 12.7% (*n* = 33) widowed; 73% (*n* = 189) of the subjects had children. 39% (*n* = 101) were high school graduates, 23.9% (*n* = 62) had a master degree, 23.6% (*n* = 61) undergone vocational training, 10.4% (*n* = 27) had a bachelor degree, and 3.1% (*n* = 8) had primary school education. In terms of income, only 5% (*n* = 13) declared to earn more than 5,000 PLN a month (ca. 1100 Euro); 12%(*n* = 31) earned up to 5,000 PLN a month, 31.7% (*n* = 82) up to 3,000 PLN, 35.9%, *n* = 93 up to 2,000 PLN, and 15.4% (*n* = 40) as little as less than a 1,000 PLN (ca. 200 Euro).

**Figure 1:**
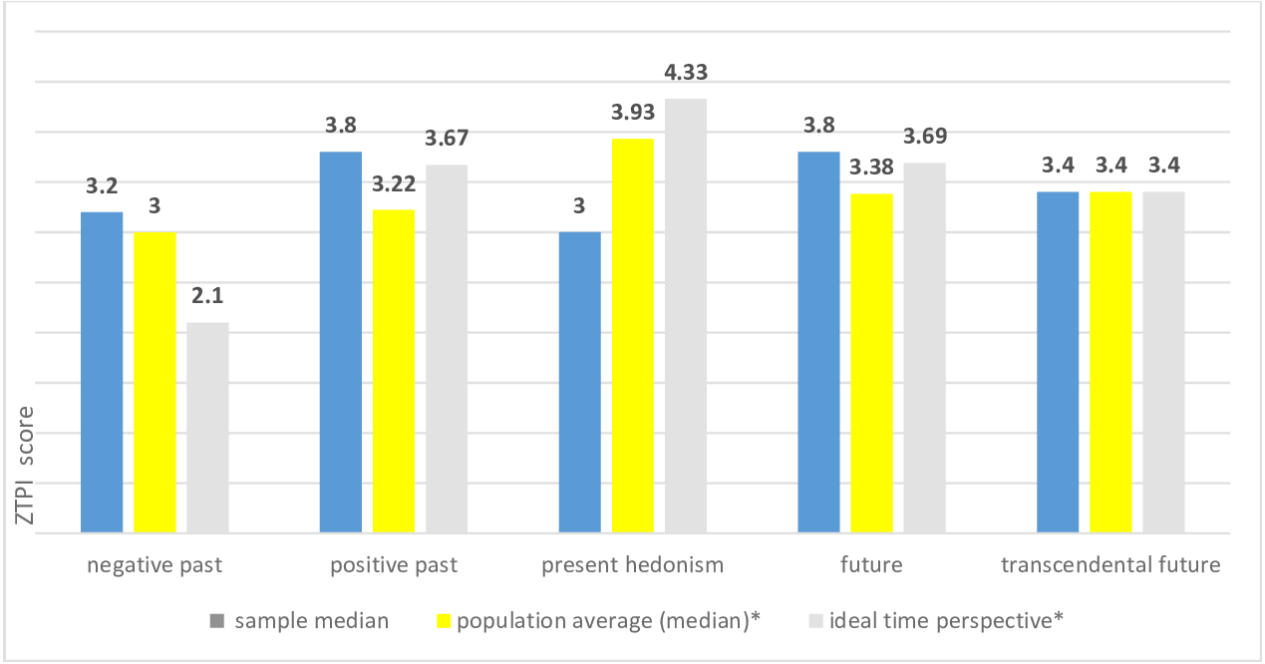
The sample time perspective scores compared to the general population score and ideal time perspective construct. Data source: http://www.thetimeparadox.com.

The patients’ time perspectives (ZTPI and TTPI mean scores) were: negative past 3.13 (Me 3.2, SD 0.871), positive past 3.69 (Me 3.8, SD 0.836), present hedonism 3.08 (Me 3.0, SD 0.811), future 3.77 (Me 3.8, SD 0.835), and transcendental future 3.4 (Me 3.4, SD 0.835). Figure 1 presents this sample median ZTPI and TTPI scores compared with general population median based on international online survey source: negative past among cancer patients is slightly higher than the general population, present hedonism is lower, and transcendental future the same. Interestingly, past positive and future perspectives of cancer patients are not only higher than the general population median but also higher than what Zimbardo and Boyd normatively consider an ideal score. It is likely because due to their focus on treatment, cancer patients are increasingly considering future scenarios.

Only selected temporal perspectives of oncological patients are dependent upon sociodemographic variables in a statistically significant way (see Table 1). Women score higher than men in transcendental future (3.52 *vs.* 3.15, *p* = 0.002), and so do believers than nonbelievers (3.57 *vs.* 2.71, *p* < 0.0001). Nonbelievers also have higher negative past perspective scores (3.32 *vs.* 3.08, *p* = 0.046). However, one must remember that the sample consisted mostly of believers (79.9%). In addition, the marital status differentiates the present hedonism perspective, which has the highest score among widowed patients (3.47) and the lowest in divorcees (2.72). However, statistically significant differences are observed between widowed and divorced patients (3.47 *vs.* 2.72, *p* = 0.004) and between widowed and in a relationship (3.47 *vs.* 3.01, *p* = 0.046). Other sociodemographic variables such as place of residence (rural/urban), professional status, income or having children, as well as the type of cancer do not have any links with the temporal perspectives (*p* > 0.05).

**Table 1:** Differences in temporal perspectives (means and medians) between selected socio-demographic groups (*N* = 259). The table contains differences in time perspectives between selected socio-demographic groups.

	**Male (Me)**	**Female (Me)**	**Believer (Me)**	**Nonbeliever (Me)**	**Single (Me)**	**In relationship (Me)**	**Divorced (Me)**	**Widowed (Me)**
**NP**	3.08 (3.20)	3.16 (3.20)	3.08 (3.00)^**^	3.32 (3.40)^**^	3.31 (3.40)	3.06 (3.00)	3.28 (3.40)	3.13 (3.20)
**PP**	3.59 (3.70)	3.74 (3.80)	3.73 (4.00)	3.53 (3.60)	3.5 (3.40)	3.72 (4.00)	3.53 (3.60)	3.9 (4.00)
**PH**	2.96 (3.00)	3.14 (3.20)	3.08 (3.00)	3.10 (3.00)	3.25 (3.20)^****^	3.01 (3.00)^****^	2.72 (2.60)^****^	3.47 (3.40)^****^
**F**	3.66 (3.60)	3.82 (4.00)	3.77 (3.80)	3.77 (3.80)	3.64 (3.80)	3.75 (3.80)	4.02 (4.00)	3.84 (3.80)
**TF**	3.15 (3.25)^*^	3.52 (3.50)^*^	3.57 (3.60)^***^	2.71 (2.70)^***^	3.46 (3.50)	3.43 (3.50)	3.10 (3.10)	3.38 (3.30)

**Divorced vs. in relationship**	**Divorced vs. single**	**Divorced vs. widowed**	**In relationship vs. single**	**In relationship vs. widowed**	**Single vs. widowed**
*p* > 0.05	*p* > 0.05	***p*** = ***0.004***	*p* > 0.05	***p*** = ***0.046***	*p* > 0.05

**Notes.**

* Mann–Whitney *U* = 5724.0, *n*1 = 86, *n*2 = 259, *p* = 0.002.

** Mann–Whitney *U* = 4376.0, *n*1 = 207, *n*2 = 52, *p* = 0.046.

*** Mann–Whitney *U* = 2350.0, *n*1 = 207, *n*2 = 52, *p* < 0.0001.

**** Kruskal-Wallis *H* test *χ*2 = 14,046, *df* = 3, *p* = 0.003 **** *Post hoc* test results.

Values in bold indicate statistical significance.

Linear time variables (age and time elapsed since diagnosis and the beginning of treatment) show no or very weak correlations with temporal perspectives (see Table 2): older people tend to have higher scores in positive past (*ρ* = 0.166, *p* < 0.05), and patients who have been treated for a longer period of time have lower transcendental future scores (*ρ* = −0.210, *p* < 0.01).

**Table 2:** Correlations between temporal perspectives and linear time variables (*N* = 259).

Linear time variables	Negative past	Positive past	Present hedonism	Future	Transcendental future
Age	0.020	**0.166** ^*^	0.092	−0.004	−0.049
Time since diagnosis	0.020	0.042	0.025	−0.026	**−0.179** ^**^
Time since the beginning of chemotherapy	−0.062	0.080	−0.014	−0.011	**−0.210** ^**^

**Notes.**

* Spearman’s *ρ*, *p* < 0.05.

** Spearman’s *ρ*, *p* < 0.01.

Values in bold indicate statistical significance.

The frequency of chemotherapy differentiates temporal perspectives of patients regarding present hedonism and transcendental future only (see Table 3). Patients in weekly treatment schemes score lower than those in triweekly schemes (2.67 *vs.* 3.18, *p* = 0.017) and those not in chemo (2.67 *vs.* 3.20, *p* = 0.009). Patients not in chemo also score higher in transcendental future than patients in biweekly (3.55 *vs.* 3.29, *p* = 0.034) and triweekly (3.55 *vs.* 3.26, *p* = 0.016) treatment.

**Table 3:** The frequency of chemotherapy and temporal perspectives with *post hoc* test results.

	**Chemo1** **(Me)**	**Chemo2** **(Me)**	**Chemo3** **(Me)**	**Not in chemo** **(Me)**
NP	2.94 (2.80)	3.19 (3.20)	3.24 (3.20)	3.06 (3.20)
PP	3.56 (3.60)	3.64 (3.60)	3.71 (4.00)	3.76 (3.90)
PH^*^	**2.67 (2.60)**	**3.03 (3.00)**	**3.18 (3.20)**	**3.20 (3.20)**
F	3.65 (3.80)	3.69 (3.80)	3.74 (4.00)	3.89 (4.00)
TF^**^	**3.53 (3.65)**	**3.29 (3.35)**	**3.26 (3.20)**	**3.55 (3.60)**

	Chemo1 vs. Chemo2	Chemo1 vs. Chemo3	Chemo1 vs. Not in chemo	Chemo2 vs. Chemo3	Chemo2 vs. Not in chemo	Chemo3 vs. Not in chemo
PH	*p* > 0.05	***p*** = ***0.017***	***p*** = ***0.009***	*p* > 0.05	*p* > 0.05	*p* > 0.05
TF	*p* > 0.05	*p* > 0.05	*p* > 0.05	*p* > 0.05	***p*** = ***0.034***	***p*** = ***0.016***

**Notes.**

* Kruskal-Wallis *H* test *χ*2 = 11,244, *df* = 3, *p* = 0.01.

** Kruskal-Wallis *H* test *χ*2 = 8,081, *df* = 3, *p* = 0.044; *post hoc* test results.

Values in bold indicate statistical significance.

## Discussion

This study concerned the relationship between different dimensions of time in cancer patients. None of the previous quantitative studies tracked the influence of either time of treatment or the rhythm of chemotherapy on temporal perspectives as defined by the ZTPI (Nozari, Janbabai & Dousti, 2013; Kroyan, Gurova & Ippolitova, 2019; Furmańska et al., 2019). Overall, the study found that linear time and the rhythm of chemotherapy are less important than hypothesized in terms of their impact on temporal perspectives. The rhythm of treatment appears more significant than linear time in the sense that time since the beginning of diagnosis and treatment only weakly correlates with the transcendental future perspective. In contrast, the rhythm affects the transcendental future and present hedonistic perspective. Weekly treatment is clearly more disturbing in terms of lowering the focus on everyday pleasures than the triweekly one, and than no chemotherapeutic treatment, which finds a common sense explanation in feeling worse with more frequent chemo. Also, patients not in chemo score significantly higher in transcendental future than patients in biweekly and triweekly treatment schemes. Still, overall, the differences between frequencies are of minor relevance.

Nevertheless, some of the cancer patients’ temporal perspective scores show how devastating the chemotherapy process is, both somatically and psychologically (Strąg-Lemanowicz & Leppert, 2014). Patients tend to mildly lose faith in the course of oncological treatment regardless of their age—future studies should verify and explore the nature of this shift in temporal perspective in more detail. In particular, they should aim to find whether it can be understood as an aspect of psychological coping strategy. Personal faith could be a source of strength for those in treatment, especially at its earlier stage, and strong faith in the afterlife could hypothetically instantiate spiritual coping strategies (Baldacchino & Draper, 2001). There is no comparison data for TTPI among cancer patients as this study is the first. Compared with other groups, however, the mean results in the sample (3.4) are still higher that TTPI scores of healthy Norwegian adults (3.19) (Skogen & Nesvåg, 2019), Portuguese students (2.84) (Ortuño & Echeverría, 2013), and Dutch people with suicidal tendencies (3.25) (Van Beek, 2013). One may speculate that this is due to the Polish population’s high level of catholic religiosity.

Furthermore, this cancer patients’ sample mean level of present hedonism is low (3.08) compared with other studies. Research performed in Iran (Nozari, Janbabai & Dousti, 2013) on 87 breast and digestive system cancer patients revealed present hedonism to be comparable (3.06), but a study from Russia on 50 reproductive system cancer patients found notably higher levels of hedonism (3.46) (Kroyan, Gurova & Ippolitova, 2019), and another Polish study of 150 patients of various cancers a much higher one (3.60) (Furmańska et al., 2019). However, none of the abovementioned studies included information about whether patients were in chemotherapy, which is likely the reason for the current sample’s lowered scores on present hedonism. This sample is also the biggest of the aforementioned. Only a study of families of cancer patients revealed a lower (2.99) level of this dimension (Wakefield et al., 2010). In a sample of diabetes patients, hedonism was only slightly higher (3.09) (Nozari, Janbabai & Dousti, 2013).

The study also found that divorced patients have the lowest level of hedonism (2.72) and widowed ones the highest (3.47). On the other hand, Wakefield et al. (2010) showed that single and divorced people with a strong history of cancer in their families have the highest scores of the hedonistic present. However, the hedonism aspect is not affected when it comes to age, sex, or family status. While treating a single patient it is therefore highly recommended to determine her/his marital status. Especially those who are under intense weekly treatment and divorced should be carefully observed for signs of decreased quality of life. On the other hand, widowed patients are likely to enjoy life the most since they do not need to care and worry about family.

On a more positive side, the study showed very high past positive and future perspective scores among cancer patients, with median values higher than the general population and the ideal scores proposed by Zimbardo and Boyd. Since no demographic variables differentiate the past positive and future ZTPI scores, cancer treatment likely unifies these temporal perspectives. One may speculate that cancer experience annihilates any preexisting differences in temporal perspectives between different sociodemographic groups, which is also supported by Wakefield et al. (2010). Regarding the future, it appears that since patients tend to focus their energy on recovery, this temporal dimension becomes idealized. Such overfocus on positive past and the future can be interpreted as a coping mechanism that helps to maintain motivation to fight the disease.

Regarding recommendations for cancer care, it appears that time perspectives among cancer patients are disproportionate and somehow stiffened. This is potentially of great importance since balanced and proportionately developed perspectives, including the ability to flexibly switch between them, are considered a predictor of well-being (Fuentes et al., 2022). Results of this study indicate that the space of possible therapeutic intervention concerns present hedonism and transcendental future. Clinicians and nurses should pay extra attention to the possible indicators of everyday pleasures appearing unreachable to the patients as well as to the arousal of their existential concerns—especially in patients undergoing long-lasting chemotherapy and being in more intensive treatment, regardless of their age and social background. While planning interventions, psychologists and psychotherapists should take the specificity of cancer patients’ temporal experience into consideration—help should not be limited to supportive aid, but extended to reframing the patients’ temporal perspective and merging their sense of temporal continuity. Doing so may support restoring the balance between time perspectives and increase the quality of life during recovery.

Patients in weekly chemotherapy with the most impoverished hedonism may need help in retaining their capabilities to focus more on the pleasures of everyday life. This may potentially enhance their positive coping strategies, of which present hedonism is recognized as an important constituent (Furmańska et al., 2019). Among the foremost available and effective psychotherapeutic tools is mindfulness, which can be used either as a trans-modal technique or introduced as a complete psychotherapeutic approach, namely Mindfulness-based Therapy (Khoury et al., 2013).

Moreover, as chemotherapy lowers transcendental future, introducing techniques centered on the image of the afterlife may be needed at times for believers. This can be achieved through consultation with a patient’s spiritual guide or through psychological counseling. Cognitive-existential psychotherapy (Kissane et al., 2003) and meaning-centered psychotherapy (Breitbart et al., 2018) are among the approaches that proved their efficacy in managing cancer patients’ existential concerns. Nevertheless, saying that every cancer patient requires psychotherapy would be a large oversimplification.

## Conclusions

This study hypothesized that the length and frequency of chemotherapy affect patients’ temporal perspectives concerning their past, present, and future as measured by ZTPI and TTPI. It was found that patients only slightly tend to lose faith alongside the course of oncological treatment regardless of their age, that some frequencies of chemotherapy statistically significantly differentiate present hedonistic and transcendental future perspectives, and that being in chemo lowers the levels of hedonism and faith. Nevertheless, the variations of treatment rhythm were found less relevant than predicted. Furthermore, the study found relatively high levels of hedonism among widowed patients, and very high past positive and future perspective scores in the whole population. The latter presumably stems from increased efforts focused on recovery. Since most sociodemographic variables are of no relevance, cancer experience likely unifies temporal perspectives among people of different backgrounds.

The major limitation of this study is that patients were approached at different stages of their illness and at different moments during their current chemo cycle. The study is, therefore, cross-sectional regarding the temporal variables of linear and circular time. In addition, since only the duration of illness was considered, the lack of knowledge regarding the cancer stage is also a limitation. Furthermore, the study did not monitor the possible mitigating effects of drugs, which could have had an impact on temporal perspectives.

##  Supplemental Information

10.7717/peerj.14486/supp-1 Data S1 All the data obtained through questionnaires

## Additional Information and Declarations

### Data Availability

The following information was supplied regarding data availability: The raw data is available in the Supplemental Files. The questionnaire is open access and available at: https://www.thetimeparadox.com/surveys/. The questionnaire is also published in a book: Zimbardo P, & Boyd J. (2008). The time paradox: the new psychology of time that will change your life. New York: Simon and Schuster. 102–118.
